# Treatment outcomes and characteristics of HIV-2 patients compared to HIV-1 patients on an NNRTI-based first line art at the adult infectious diseases centre of the University Teaching Hospital (UTH) in Lusaka

**DOI:** 10.11604/pamj.2021.40.231.25149

**Published:** 2021-12-16

**Authors:** Anita Thandiwe Bhebhe, Gershom Chongwe, Given Moonga

**Affiliations:** 1 Department of Epidemiology and Biostatistics, School of Public Health, University of Zambia, Lusaka, Zambia,; 2 Tropical Diseases Research Centre, Ndola, Zambia,; 3 Center for International Health, Hospital of the Ludwig Maximilian University, Munich, Germany

**Keywords:** HIV-2, NNRTI, immunological, virological treatment outcome

## Abstract

**Introduction:**

the focus of antiretroviral therapy (ART) in Zambia has been on HIV-1. However, some patients are infected with HIV-2 or both. HIV-2 is resistant to non-nucleoside reverse transcriptase inhibitors (NNRTIs), drugs used for HIV-1. Therefore, this study sought to determine the seroprevalence of HIV-2 or dual infection in HIV infected individuals and compare the treatment outcomes associated with HIV subtype in patients taking NNRTI-based first line cART at the University Teaching Hospitals (UTH).

**Methods:**

this was a cross- sectional study, we collected data from the Virological Impact of Switching from Efavirenz and Nevirapine based first-line cART regimens to Dolutegravir (VISEND) study being conducted at UTH. Ninety six individuals were included in the study. Descriptive and inferential statistics were performed. Logistic regression was used to assess the relationship between treatment outcomes and HIV type.

**Results:**

the proportion of HIV 1 and 2 co-infected patients was 5.2% (95% CI 2%-12%). The mean age was 46 years ± 2 years with 60 (62.5%) being females. The median viral load was 1.3 log 10 copies/ml, IQR 0-1.7 log 10 copies/ml and the median absolute CD4+ T cell count increased from 231 to 463 cells/mm^3^ (p < 0.001) after being on cART for one year or more. The study did not report any associations between treatment outcomes and HIV type (p > 0.05).

**Conclusion:**

there is a small proportion of patients that are HIV 1 and 2 co-infected but are on an NNRTI-based cART regimen, drugs that are not active against HIV-2. This, however, does not seem to significantly affect the patient´s virological or immunological treatment outcome.

## Introduction

HIV continues to be a major global public health issue that has claimed more than 35 million lives so far. The African region is the most affected region with 25.6 million people living with HIV in 2016. It also accounts for approximately two-thirds of the global total of new HIV infections [1]. In 2016, Zambia had 59 thousand new HIV infections and 21 thousand AIDS-related deaths. There were 1.2 million people living with HIV and among these, 65% were accessing combination antiretroviral therapy [2]

HIV-1 is the most predominant type of HIV, accounting for approximately 95 per cent of all HIV infections worldwide. HIV-2 is less common than HIV-1, and is predominantly found in West Africa. It is also less infectious than HIV-1 and although HIV-2 responds less predictably to combination antiretroviral therapy (cART) [3], it progresses more slowly leading to fewer deaths [4]. The two retroviruses are related and in regions where both infections are endemic, HIV 1 and 2 dual infection can occur (De Silva *et al*, 2010) [5]. Although they are related, HIV-1 and HIV-2 are genetically distinct with only a 55 per cent sequence similarity [6].

In order to have a diagnosis that is HIV type-specific, an initial HIV test using an HIV-1/HIV-2 antigen/antibody combination immunoassay and subsequent testing using an HIV-1/HIV-2 antibody differentiation immunoassay is recommended [7]. Studies suggest that commercially available HIV-1 viral load assays do not reliably detect HIV-2 [8,9]. The best strategy with which to treat HIV-2 has been less clearly defined than HIV-1. Although most antiretroviral drugs are active against HIV-2, it is resistant to non-nucleoside reverse transcriptase inhibitors (NNRTIs) such as Nevirapine and Efavirenz [10]. In Zambia, clinicians are therefore advised to include two nucleoside reverse transcriptase inhibitors (NRTIs) with dolutegravir (an intergrase inhibitor) or Lopinavir/ritonavir (a protease inhibitor) when prescribing cART for HIV-2 mono-infected or HIV 1 and 2 co-infected individuals and not prescribe NNRTIs or the PI Atazanavir as part of a cART regimen against HIV-2 mono-infection [11].

Lastly, in order to determine the performance of cART in a patient, viral load (the most preferred tool), CD4+ T cell count and clinical monitoring may be used [3]. If a patient´s viral load is persistently above 1,000 copies/ml even after two consecutive measurements within a three-month interval, with adherence counseling between measurements, and the patient has been on cART for at least 6 months, it can be concluded that treatment failure has occurred in this patient. Also, studies show that the risk of transmitting the HIV virus or progressing to AIDS is relatively low when the viral load is less than 1,000 copies/ml [12]. For most patients, by the end of their first year on cART, an adequate response is defined as an increase in CD4+ T cell count of at least 50-150 cells/ml [13]. To the best of our knowledge we did not find any published information on HIV-2 seroprevalence in Zambia or the consequences of misdiagnosis of HIV-2 as HIV-1 and subsequent treatment of HIV-2 with drugs that are not recommended for HIV-2. The study aimed at determining the seroprevalence of HIV-2 and dual infection in HIV infected individuals and comparing the treatment outcomes associated with HIV subtype in patients on NNRTI based first line cART at AIDC of UTH.

## Methods

**Study setting:** the University Teaching Hospital (UTH) is located in Zambia´s capital city, Lusaka. It has a catchment population of approximately 2 million. The facility has five hospitals, namely; Adult Hospital, Women and Newborn Hospital, Eye Hospital, Children´s Hospital, and the Cancer Diseases Hospital (CDH). For this study, we focused on the Adult Hospital where there is the Adult Infectious Diseases Centre (AIDC) which is the country´s main referral centre for HIV/AIDS services. AIDC offers first-line, second-line and third-line (advanced) treatment of HIV/AIDS. In this study, we used pre-screening and screening data collected from patients at the AIDC that were screened for possible enrollment into the VISEND (Virological Impact of Switching from Efavirenz and Nevirapine based first line ART Regimens to Dolutegravir). The patients were on Zambia´s first-line treatment of HIV.

**Study design, sample size and sampling technique:** in this cross-sectional study, we adopted the proportion of HIV-2 among HIV patients as reported in a study conducted in Guinea Bissau [14] for our sample size calculation. The proportion of HIV-2 decreased from 4.4% in 2006 to 2.8% in 2016 with an age-adjusted and sex-adjusted prevalence ratio (aPR) of 0.71 [95% confidence interval (CI)=0.59-0.85]. With the above assumption, the minimum sample size needed was estimated as 96 individuals. Systematic sampling method was employed, selecting every second participant from a database of 152 participants that met the study criteria until the minimum sample size was achieved.

**Data collection:** the study obtained information on 766 participants from the VISEND study between May 2019 and October 2019. Of these participants, 538 had an HIV 1 and 2 differentiation test result, and 152 had complete and usable data from the UTH SmartCare system. A data extraction tool was used to collect this data. Demographic factors, socioeconomic factors, and ART start date were extracted from the UTH SmartCare information system. The demographic factors of interest were: sex, age (as a continuous variable) and marital status. Two markers of socioeconomic status were considered: place of residence (low cost, medium cost and high cost) and highest education level attained at the start of ART. The age of the participants was further categorized as a young adult (18-29 years), adult (30-44 years), middle-aged (45-64 years) and elderly (65 years and above). The viral load for all participants was obtained from the VISEND data set. Participants with a viral load of fewer than 1,000 copies/ml of blood were categorized as virologically suppressed whereas those with a viral load of 1,000 or more copies/ml were categorized as virologically unsuppressed. The baseline CD4+ T cell count (cells/mm^3^) of the participants was measured retrospectively approximately one year before the current CD4+ T cell count which was obtained from the VISEND study. A reduction, stagnation or increment of less than 100 CD4+ T cells/mm^3^ was considered to be a poor immunological outcome while an increment of 100 CD4+ T cells/mm^3^ or more was considered to be a good immunological outcome. Since the HIV-2 differentiation test in Zambia was introduced after the emergence of the 2016 ART guidelines, the participants were categorized into two groups; one group was composed of participants that started their ART before 2016 and the other was composed of participants that started their ART from 2016 onward.

**Data analysis:** the VISEND data set did not record any participant with HIV-2 mono-infection. Therefore, in this study, there was one dichotomous outcome variable, namely; HIV type (HIV-1 mono-infection or HIV 1 and 2 co-infection). Data collected from the extraction tool was sorted and tabulated. Data were coded and entered into excel for data validation. The excel spreadsheet was then exported to STATA version 14 (Stata Corporation, College Station, TX, USA) for further cleaning and analysis. Categorical variables were reported as numbers and percentages. Pearson´s chi-square test was used to assess statistical differences between categorical variables with significance level set at p<0.05 and 95% confidence interval. Normally distributed continuous data were reported as means and standard deviations. The Shapiro-Wilk test was used to test for normality. In this study, only age was normally distributed (Shapiro-Wilk test expressed a p-value greater than 0.05). Other continuous data were reported using medians and interquartile ranges. Additionally, the Wilcoxon rank-sum test was used to compare the medians of the baseline and the current CD4+ T cell counts. Univariable and multivariable analyses were performed using logistic regression. Associations between participant characteristics and HIV type were evaluated using odds ratios and their associated 95% confidence intervals. Variables that expressed co-linearity were removed from the final model. A p-value of less than 0.05 was considered significant.

**Ethical considerations:** permission to obtain data from the VISEND study was sought and obtained from its principal investigator. Ethical approval to undertake the study was sought from University of Zambia Biomedical Research Ethics Committee (UNZABREC), reference number 025-08-18.

The study ensured that participants were randomly selected, thereby giving every patient an equal and fair chance of selection in the study. Additionally, all patient files were de-identified by assigning a unique code to each patient file for the purpose of confidentiality. Finally, data that was acquired from this research was used only for the purposes of this study. The data obtained was kept on a computer-accessible only by a password and by the investigator.

There was no direct benefit to the participants in this study. However, HIV-2 patients whose cART regimen contained Nevirapine or Efavirenz and had not been switched to the correct regimen at the time of the study were followed up for possible regimen change.

## Results

A total of 96 HIV infected individuals were considered for this study after eliminating missing and non-usable data. Among the participants, the mean age was 46 years ± 2 years with 60 (62.5%) being females. The median ART start year was 2013, interquartile range (IQR) 2009-2016, the median baseline CD4+ T cell count was 231 cells/mm^3^, IQR 133-422 cells/mm^3^ and the median viral load was 1.3 log 10 copies/ml, IQR 0-1.7 log 10 copies/ml. The median absolute CD4+ T cell count increased from 231 to 463 cells/mm^3^ (p < 0.001) (Table 1). In this sample, it was discovered that five (5.2%) individuals had HIV 1 and 2 co-infection and none had HIV-2 mono-infection. The prevalence of HIV 1 and 2 co-infection was therefore 5.2% (95% confidence interval 2%-12%).

**Table 1:** demographic and clinical characteristics of participants by HIV type (n=96)

Variable	Total n (%)	HIV-1 n (%)	HIV 1 & 2 n (%)	P value
**Total**	96 (100)	91 (94.8)	5 (5.2)	-
**Sex**				
Female	60 (62.5)	58(63.7)	2 (40.0)	
Male	36 (37.5)	33 (36.3)	3 (60.0)	0.29
**Age (yr)**				
18-29	3 (3.1)	2 (2.2)	1 (20.0)	
30-44	38 (39.6)	37 (40.7)	1 (20.0)	
45-64	50 (52.1)	48 (52.7)	2 (40.0)	
65+	5 (5.2)	4 (4.4)	1 (20.0)	0.05
Mean age 46.4, SD 9.5				
**Marital Status**				
Never married	9 (9.4)	9 (9.9)	-	
Married	54 (56.2)	54 (59.3)	5 (100)	
Divorced/ Separated	19 (19.8)	14 (15.4)	-	
Widowed	14 (14.6)	14 (15.4)	-	0.35
**Place of Residence**				
Low cost	49 (51.0)	45 (49.4)	4 (80.0)	
Medium cost	35 (36.5)	34 (37.4)	1 (20.0)	
High cost	12 (12.5)	12 (13.2)	-	0.38
**Highest Education Level**				
No education	4 (4.2)	4 (4.4)	-	
Primary	18 (18.7)	16 (17.6)	2 (40.0)	
Secondary	55 (57.3)	53 (58.2)	2 (40.0)	
Tertiary	19 (19.8)	18 (19.8)	1 (20.0)	0.62
ART Start Year *				
<2016	65 (68.7)	64 (70.3)	2 (40.0)	
2016+	30 (31.3)	27 (29.7)	3 (60.0)	0.15
**Median ART start year 2013, IQR 7**				
Immunological outcome				
Poor	33 (33.3)	30 (33.0)	2 (40.0)	
Good	64 (66.7)	61 (67.0)	3 (60.0)	0.75
**Median baseline CD4+ 231, IQR 289**				
**Median current CD4+ 463, IQR 363**				
**Viral Load**				
<1000	84 (87.5)	80 (87.9)	4 (80.0)	
1000+	12 (12.5)	11 (12.1)	1 (20.0)	0.60
Median viral load 1.3 log 10, IQR 1.7				

*All study participants were on an NNRTI based first line ART, that is Tenofovir/ Lamivudine/ Efavirenz or Tenofovir/ Lamivudne/ Nevirapine

As shown in Table 1, the majority of study participants were in the age ranges of 30-44 years and 45-64 years [38 (39.6%) and 50 (52.1%), respectively]. Most of these patients were married [54 (56.2%)], lived in low cost and medium cost residential areas [49 (51.0%) and 35 (36.5%), respectively], and had attained at least secondary school level of education [55 (57.3%)]. It is also important to note that all five of the dually positive patients were married, three were male and four lived in a low cost residential area.

The total proportion of patients that started taking their cART drugs before the release of the 2016 guidelines was 65 (68.7%). Among those with HIV-1 mono-infection, 64 (70.3%) started their cART before 2016 while two (40.0%) of the dually positive patients started their ART before 2016. Additionally, 64 (66.7%) patients had a good immune response to cART as observed from a comparison between their baseline and their current CD4+ T cell count with an HIV-1 proportion of 61 (67.0%) and an HIV 1 and 2 proportion of three (60.0%). Most of the study participants [84 (87.5%)] were virally suppressed (had a viral load of less than 1,000 copies/ ml). Of those who were HIV-1 mono-infected, 87.9% were virally suppressed while four (80.0%) of the HIV 1 and 2 co-infected were virally suppressed.

Logistic regression was used to analyze relationships between the dependent variable (HIV type) and explanatory variables (sex, age, ART start year, immune response, and virological suppression status). The model allowed estimating the probability of one having HIV-1 mono-infection or HIV 1 and 2 co-infection. Results show both unadjusted and adjusted estimates as depicted in Table 2. In both the unadjusted and adjusted analyses, all of the independent variables showed insignificant associations with HIV type. Additionally, the wide confidence intervals suggest that the associations made occurred due to chance. This study found that immunological and virological treatment outcomes and other patient characteristics of patients taking NNRTI-based cART at UTH were not HIV type-specific.

**Table 2:** treatment outcomes and characteristics of participants according to HIV type (n=96)

Description	Crude odds ratio (95% CI)	P value	Adjusted odds ratio (95% CI)	P value
**Sex**				
Female	Ref			
Male	2.6 (0.42-16.59)	0.30	6.77 (0.51-89.05)	0.15
**Age**				
18-29	Ref			
30-44	0.05 (0.00-1.22)	0.07	0.05 (0.00-1.28)	0.07
45-64	0.08 (0.01-1.35)	0.08	0.12 (0.00-2.70)	0.18
65+	0.50 (0.02-12.9)	0.68	0.69 (0.02-14.51)	0.83
**ART start year**				
<2016	Ref			
2016+	3.56 (0.56-22.49)	0.18	4.66 (0.24-91.09)	0.31
**Immune response**				
Poor	Ref			
Good	0.74 (0.12-4.65)	0.75	1.62 (0.16-16.15)	0.68
**Viral Load**				
<1,000	Ref			
1,000+	1.8 (0.19-17.78)	0.61	1.83 (0.13-26.65)	0.66

## Discussion

In this study, we aimed at determining the seroprevalence of HIV-2 and dual infection in HIV infected individuals, and associated factors. We also aimed at comparing the treatment outcomes associated with HIV subtype in HIV-infected patients on non-nucleoside reverse transcriptase inhibitor (NNRTI)-based first line combination antiretroviral therapy (cART). Currently, there is very little evidence of the presence of HIV-2 in Zambia prior to the nation´s 2016 guidelines. In fact, three decades ago, one study could not find any HIV-2 antibodies in 6 Central African nations, one of which was Zambia [15]. Evidently, this is no longer the case because according to our study, the seroprevalence of HIV 1 and 2 co-infection in HIV patients taking an NNRTI-based first line cART regimen was found to be 5.2 per cent. This is in consensus with a study conducted in Guinea Bissau where researchers discovered a seroprevalence of HIV 1 and 2 co-infection of 6 per cent [16].

Our study could not find a statistically significant difference in the distribution of sex according to HIV-type. This was inconsistent with findings from Guinea Bissau where women were reportedly more likely than men to be infected with HIV-2, or dually infected [17]. The reasons for this difference are assumed to be due to the longer period of time that the data for the above study was carried out as compared to our study which was a cross-sectional study. Our study findings also found a negative association between age ranges of adult to middle aged individuals, and HIV-2 infection when compared with young adults but did not reach statistical significance. This was inconsistent with findings from Guinea Bissau where HIV-2 prevalence peaked in men (60-69 years) and women (50-59 years) [18]. This pattern could be due to differential mortality for HIV-2 infected individuals or to a cohort effect for a generation who were sexually active at the time of the war of independence in the 1960s and early 1970s in Bissau. The Bissau study participants were also all 50 years and above.

This study also revealed that 40 per cent of the dually positive patients started their ART before 2016. It is unclear as to whether these patients were HIV-1, HIV-2 or HIV 1 and 2 co-infected at the point of ART initiation. Over half of the HIV-1 individuals had a good immunological treatment outcome which was comparable to those who were HIV 1 and 2 co-infected. There was a 2-fold increase in the median CD4+ T cells count. A study in Kenya reported similar findings where a median CD4+ T cell count increase of 210 cells/mm^3^ was reported in patients on an NNRTI-based cART regimen [19] and another study using the Swiss HIV cohort also reported a 2-fold increase in the mean CD4+ T cell count [20]. In Ethiopia, a comparison between two groups taking NNRTIs discovered that the Nevirapine arm had a mean CD4+ T cell increase of 215 cells/mm^3^ while that of the Efavirenz arm had a comparable increment of 205 cells/mm^3^ [21].

After adjusting for sex, age, ART start year and viral suppression, our study demonstrated a positive association between a good immune response and the presence of HIV-2. This finding was, however, statistically insignificant. A different report was received from patients in five West African countries on an NNRTI-based regimen, where the mean CD4+ T cell count change in 12 months was significantly lower in HIV-2 and dually positive patients compared to HIV-1 patients [22]. Therefore, perhaps repeating the study with a larger sample size would yield more conclusive associations between a patient being infected with HIV-2 on an NNRTI-based regimen and their immunological treatment outcome. Impressively, the majority of our study participants (whether HIV-1 mono-infected or HIV 1 and 2 co-infected) were HIV-1 RNA virally suppressed. Similar findings were reported in Ethiopia in HIV patients on an NNRTI-based regimen [21]. An association between viral suppression and HIV type could not be established because our findings were statistically insignificant when both the Pearson´s chi-square test and simple logistic regression were employed.

The median viral load in our sample was quite different from that of Kenyan children with advanced HIV-1 disease where the median viral load was 2.2 log 10 copies/ml [19]. The reasons for the difference could be that the viral loads in the Kenyan study were measured after 6 months of therapy with an NNRTI-containing regimen as opposed to our study were most patients were on cART for more than 1 year. Additionally, a study in Thailand discovered that approximately 75% of children had HIV-1 RNA viral load of less than 1.7 log 10 copies/ml at 96 weeks of NNRTI based therapy [23]. Two major limitations worth noting were observed in this study. Firstly, only a minimum sample size was achieved, therefore it may have limited the scope of the research. Secondly, the choice of variables included in the study was limited by the use of medical records. Therefore, some variables that may explain treatment outcomes according to HIV type such as the patient´s HIV type at the point of ART initiation were not collected.

Despite these limitations, this study has brought to light interesting aspects that can help improve treatment options for patients whose HIV type is unknown and provide better treatment outcomes for HIV-infected individuals, regardless of HIV type. Additionally, studies have shown that with disease progression, HIV-1 out-competes HIV-2 in dually positive patients [24,25]. This suggests that in-vivo immunodeficiency is probably driven by HIV-1 in co-infected patients.

## Conclusion

Our study was able to identify a small proportion of patients that are HIV 1 and 2 co-infected but are on a regimen that contains Nevirapine or Efavirenz, drugs that are not active against HIV-2. This, however, does not seem to significantly affect the patient´s virological or immunological treatment outcomes. We believe that greater priority should be given to HIV-2 research because of the unique insights this viral species can provide about the pathophysiology of HIV-1, and treatment outcomes in HIV patients regardless of subtype and cART regimen.

### What is known about this topic

- *As observed from the emergence of the 2016 cART guidelines, HIV-1 and HIV-2 infection presently exists in Zambia, including dual infection, although actual seroprevalence is currently unknown;*
- *Previous studies show that HIV-2 is resistant to non-nucleoside reverse transcriptase inhibitors, drugs that are used in the treatment of HIV-1*.

### What this study adds

- *The study determines the seroprevalence of HIV-2 or dual infection in patients taking NNRTI-based first line cART at the highest tertiary hospital in Zambia and highlights the need to periodically re-test patients that on an NNRTI-based cART regimen in order to improve adherence to national cART guidelines;*
- *The study also attempts to determine the treatment outcomes associated with HIV-2 or dual infection in this population, so as to estimate the effect of misdiagnosis of HIV-2 in HIV patients taking an NNRTI-based cART regimen*.
